# Comparing the *Bbs10* complete knockout phenotype with a specific renal epithelial knockout one highlights the link between renal defects and systemic inactivation in mice

**DOI:** 10.1186/s13630-015-0019-8

**Published:** 2015-08-13

**Authors:** Noëlle Cognard, Maria J Scerbo, Cathy Obringer, Xiangxiang Yu, Fanny Costa, Elodie Haser, Dane Le, Corinne Stoetzel, Michel J Roux, Bruno Moulin, Hélène Dollfus, Vincent Marion

**Affiliations:** Ciliopathies Modeling and Associated Therapies Team, Laboratory of Medical Genetics, National Institute for Health and Medical Research (INSERM), U1112, Université of Strasbourg, 11 rue Humann, 67085 Strasbourg, France; Service de Néphrologie-Transplantation, Nouvel Hôpital Civil, 1 place de l’Hôpital, 67091 Strasbourg, France; Institut Clinique de la Souris, Illkirch, 67400 Strasbourg, France; Service de Génétique Médicale, Institut Génétique Médicale d’Alsace, Hôpital de Hautepierre, Hôpitaux Universitaires de Strasbourg, Strasbourg, France

**Keywords:** Bardet–Biedl Syndrome, *Bbs10*, Total knockout, Retinal degeneration, Obesity, Renal epithelial-specific KO

## Abstract

**Background:**

Bardet–Biedl Syndrome (BBS) is a genetically heterogeneous ciliopathy with clinical cardinal features including retinal degeneration, obesity and renal dysfunction. To date, 20 BBS genes have been identified with *BBS10* being a major *BBS* gene found to be mutated in almost 20 percent of all BBS patients worldwide. It codes for the BBS10 protein which forms part of a chaperone complex localized at the basal body of the primary cilium. Renal dysfunction in BBS patients is one of the major causes of morbidity in human patients and is associated initially with urinary concentration defects related to water reabsorption impairment in renal epithelial cells. The aim of this study was to study and compare the impact of a total *Bbs10* inactivation (*Bbs10*^−*/*−^) with that of a specific renal epithelial cells inactivation (*Bbs10* ^*fl/fl*^*; Cdh16*-*Cre*^+*/*−^).

**Results:**

We generated the *Bbs10*^−*/*−^*and Bbs10* ^*fl/fl*^*; Cadh16*-*Cre*^+*/*−^ mouse model and characterized them. *Bbs10*^−*/*−^ mice developed obesity, retinal degeneration, structural defects in the glomeruli, polyuria associated with high circulating arginine vasopressin (AVP) concentrations, and vacuolated, yet ciliated, renal epithelial cells. On the other hand, the *Bbs10* ^*fl/fl*^*; Cadh16*-*Cre*^+*/*−^mice displayed no detectable impairment.

**Conclusions:**

These data highlight the importance of a systemic *Bbs10* inactivation to trigger averted renal dysfunction whereas a targeted absence of BBS10 in the renal epithelium is seemingly non-deleterious.

**Electronic supplementary material:**

The online version of this article (doi:10.1186/s13630-015-0019-8) contains supplementary material, which is available to authorized users.

## Background

Bardet–Biedl syndrome (BBS) [1] is an autosomal recessive ciliopathy for which 20 genes (BBS1-20) have been identified to date. It is clinically characterized, among features, by early-onset retinitis pigmentosa (RP), obesity and renal dysfunction as well as polydactyly, RP is a rod–cone dystrophy [2], leading to progressive visual loss and blindness [3]. The connecting cilium of photoreceptors, a highly specialized primary cilium [3, 4], plays a pivotal role in intraciliary transport (ICT) between the inner and outer segments [5]. The established mechanism behind this phenotype is an excessive accumulation of protein in the inner segment (IS) of the photoreceptor due to defect in intracellular transport (ICT) [6, 7] triggering an unfolded protein response-mediated apoptosis of the photoreceptors [8–10]. In parallel, obesity develops together with the RP [2] and is associated with hyperleptinemia. The high circulating concentrations of Leptin observed both in patients [11] and mouse models [12, 13] have historically been linked to a hypothalamic defect in the regulation of food intake leading to hyperphagia and decreased energy expenditure [12]. More recently, BBS proteins have been shown to play a key role in the adipose tissue maturation and function [14] which strongly suggest that multiple pathways could be driving the BBS-obese phenotype.

Compared to RP and obesity, BBS-induced renal impairment after birth tends to display a late-onset profile as it is generally detected in the late childhood. This major cause of morbidity and mortality for the BBS patients is characterized by an initial urinary concentrating defects associated with polyuria–polydypsia [15]. Concomitantly, scores of structural abnormalities like small kidneys with normal or irregular contours and reduced parenchyma thickness, calyceal distortion, clubbing and blunting, caliectasis, presence of medullary cysts communicating or not with mildly dilated calyceal system and occasional presence of cortical cysts have been reported and they represent the full clinical spectrum of the BBS renal phenotype [16]. To explain these BBS renal phenotypes several defective signalling pathways have been incriminated such as a defective WNT signalling cascade during development [17, 18] as well as the lack of ciliary targeting of the antidiuretic hormone arginine vasopressin (AVP) isoform 2 receptor (AVPR2). In combination to these intrinsic effects of BBS inactivation, it has been reported that the occurrence of obesity was a leading cause for the presence of renal cysts in mice; an effect which was reverted when they went through food restriction [18]. Although the results highlight the difficulty in identifying the exact pathological mechanism leading to renal dysfunction in the BBS, in an attempt to understand the precise impact of BBS inactivation in the ciliated renal epithelium, we generated the *Bbs10*^−*/*−^ mice and the *Bbs10* ^*fl/fl*^*; Cadh16*-*Cre*^+*/*−^ and studied the resulting phenotypes.

## Methods

### Generation of *Bbs10* total knockout mice and *Bbs10* renal epithelial-specific knockout mice

All experimental procedures were approved by the local ethical committee of Strasbourg. See Additional file 1: Supporting information for supplementary methods. The inactivation of the *Bbs10* gene was achieved by the deletion of the exon 2. *Bbs10* ^*fl/fl*^ mice were obtained by DNA recombination, using LoxP sites flanking the 5′ and 3′ regions of the exon 2 in *Bbs10* gene in embryonic stem cells line 129OLA using a neomycin cassette as a selectable marker (Additional file 2: Figure S1). These mice were bred with Cadh16-CreDeleter mice (B6.Cg-Tg (*Cadh16*-*Cre*) 91Igr/J, stock number 012237 from Jackson). Homozygous knockout mice *Bbs10*^−*/*−^ and the control littermates *Bbs10*^+*/*+^ were obtained by mating of heterozygous *Bbs10*^+*/*−^mice. Chimeric mice were obtained on a C57/BL6 genetic background. *Bbs10* ^*fl/fl*^ mice were bred with the Ksp1.3/Cre transgenic mouse lines expressing heterozygous Cre recombinase gene under the control of a tissue-specific promoter: Ksp-Cadherin (Cadherin16), exclusively expressed in renal tubular epithelial cells [19]. The resulting *Bbs10* ^*fl/fl*^*; Cadh16*-*Cre*^+*/*−^ together with the *Bbs10*^+*/*+^*; Cadh16*-*Cre*^+*/*−^ mice were generated. All the genotypes were checked by PCR using KAPA Mouse Genotyping Kit (#KK7302, Kapa Biosystems, Woburn, Massachusetts, USA). Primer sequences used for *Bbs10* genotyping were 5′-ACA AAT ACA ATT GAT CAT CGA TGT G-3′ (forward primer) and 5′-GTT GCC TGG CTT GGG TGG CA-3′ (reverse primer), and 5′-ACC TCC CCA CTT GAA CGA GGT CT-3′ (forward) and 5′-GTT GCC TGG CTT GGG TGG CA-3′ (reverse) for WT and floxed mice. For *Cadh16 Cre* genotyping, the primer sequences used were 5′-GCA GAT CTG GCT CTC CAA AG-3′ as sense primer and 5′-AGG CAA ATT TTG GTG TAC GG-3′ as antisense primer, and 5′-CAA ATG TTG CTT GTC TGG TG-3′ (forward) and 5′-GTC AGT CGA GTG CAC AGT TT-3′ (reverse) for the internal positive control. Genotyping at the ROSA26 locus of the RosaTomato/eGFP (Gt(ROSA)26Sor^tm4(ACTB-tdTomato,−EGFP)Luo^/J, Jackson Laboratories Stock # 007576) mice was performed using the following three-primer set: *oIMR7318:* 5′-CTC TGC TGC CTC CTG GCT TCT-3′; *oIMR7319:* 5′-CGA GGC GGA TCA CAA GCA ATA-3′; *oIMR7320:* 5*′-*TCA ATG GGC GGG GGT CGT T-3′. See Additional file 1: Supporting information for supplementary methods.

## Results

### Molecular characterisation of the *Bbs10*^−*/*−^ mice

**Fig. 1:**
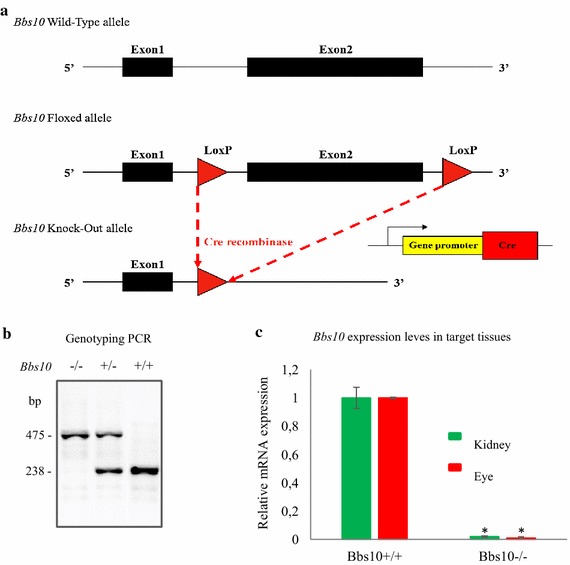
Generating the *Bbs10* ^−*/*−^ mouse model. **a** Schematic representation of the targeting strategy for *Bbs10* allele. **b** Genotyping PCR results for homozygous knock out (*Bbs10* ^−*/*−^), heterozygous (*Bbs10* ^+*/*−^), wild-type (*Bbs10* ^+*/*+^) mice. PCR bands at 475 and 238 bp correspond to the excised allele and to the natural allele, respectively. (bp base pairs). **c** Relative mRNA expression levels of *Bbs10* in kidneys and the eyes of *Bbs10* ^+*/*+^ and *Bbs10* ^−*/*−^. Reference gene *Gapdh* (n = 4 per genotype).

Using a PCR-based approach, we generated a conditional floxed mice (*Bbs10* ^*fl/fl*^) in which we flanked the exon2 on the 3′- and 5′-regions with LoxP sites (Fig. 1a and Additional file 2: Figure S1). Total Bbs10 knockout mice (*Bbs10*^−*/*−^) were then produced by breeding the *Bbs10* ^*fl/fl*^ mice with a Deleter-Cre mouse line. Following Cre-mediated excision of the *Bbs10* allele, the Cre allele was removed through breeding the resulting heterozygous *Bbs10* mice *Bbs10*^+*/*−^. The *Bbs10*^+*/*−^ mice were then used for breeding to generate the total Bbs10 knockout mice *Bbs10*^−*/*−^, and were subsequently genotyped by PCR (Fig. 1b). *Bbs10*^−*/*−^ mice display no *Bbs10* expression level in target tissues like kidney and the eyes (Fig. 1c). Bbs10^+*/*+^ and Bbs10 ^*fl/fl*^ had no differences in Bbs10 mRNA expression as can be seen in Additional file 3: Figure S2D.

### *Bbs10*^−*/*−^ mice display typical BBS-obese phenotype

**Fig. 2:**
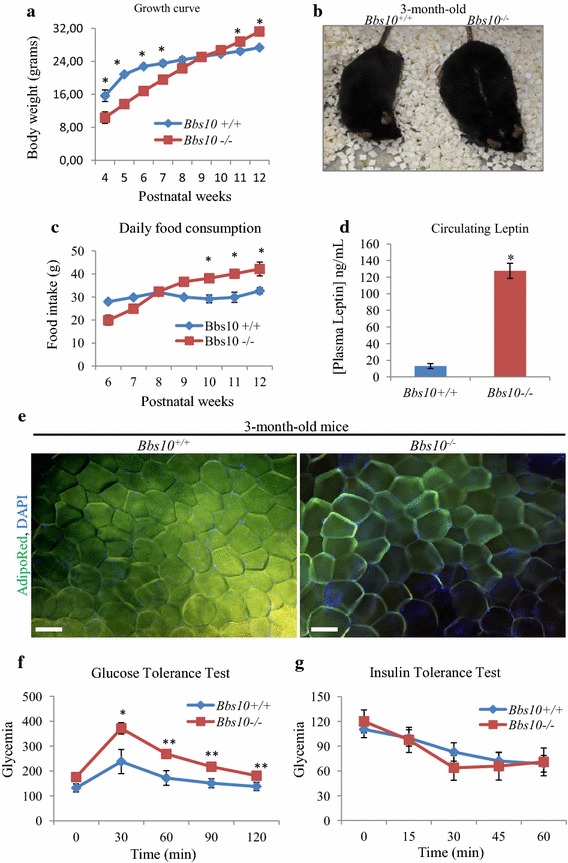
Obesity-associated phenotype in *Bbs10* ^−/−^ mice. **a** Growth curve of *Bbs10* ^+*/*+^ and *Bbs10* ^−*/*−^ (n = 8). **b** Photograph of 3-month-old *Bbs10* ^+*/*+^ and *Bbs10* ^−*/*−^ mice. **c** Food intake measurement of *Bbs10* ^+*/*+^ and *Bbs10* ^−*/*−^ mice (n = 8 per genotype). **d** Measurement of the circulating concentrations of leptin in *Bbs10* ^+*/*+^ and *Bbs10* ^−*/*−^ (n = 6 per group). **e** Pictures of AdipoRed-stained visceral adipocytes from *Bbs10* ^+*/*+^ and *Bbs10* ^−*/*−^. *Scale bar* 50 µm. Data are expressed as mean ± SEM. **f** Glucose tolerance test (GTT) and **g** insulin tolerance test (ITT) for the same mice (n = 7 per group). Values are expressed as mean ± SEM. *p < 0.05.

In the perinatal period, *Bbs10*^−*/*−^ mice exhibited a runting phenotype but recovered equivalent body weight with the control *Bbs10*^+*/*+^ littermates by 8 weeks of age. As from 8 weeks onwards, *Bbs10*^−*/*−^ mice gained more weight than the *Bbs10*^+*/*+^ (Fig. 2a) and at 3 months, the *Bbs10*^−*/*−^ mice were overweight (Fig. 2b). Food intake measurements revealed that *Bbs10*^−*/*−^ mice only develop hyperphagia at 8 weeks postnatal (Fig. 2c) and presented severe hyperleptinemia at 3 months with circulating leptin concentration of 125 ng/mL (Fig. 2d). Despite the obese phenotype, subsequent analysis examination of the visceral adipocytes showed no significant difference in cellular diameter between *Bbs10*^−*/*−^ and *Bbs10*^+*/*+^ mice (Fig. 2e). Normal glycaemic levels were observed in fasted, unchallenged animals although a delay in the rate of decrease of glucose levels was observed following glucose tolerance test (GTT) (Fig. 2f). Furthermore, insulin sensitivity was maintained in *Bbs10*^−*/*−^ as demonstrated by the insulin tolerance test (ITT) results; glucose level patterns were similar between the *Bbs10*^−*/*−^ and *Bbs10*^+*/*+^mice (Fig. 2g).

### *Bbs10*^−*/*−^ mice exhibit retinal degeneration

**Fig. 3:**
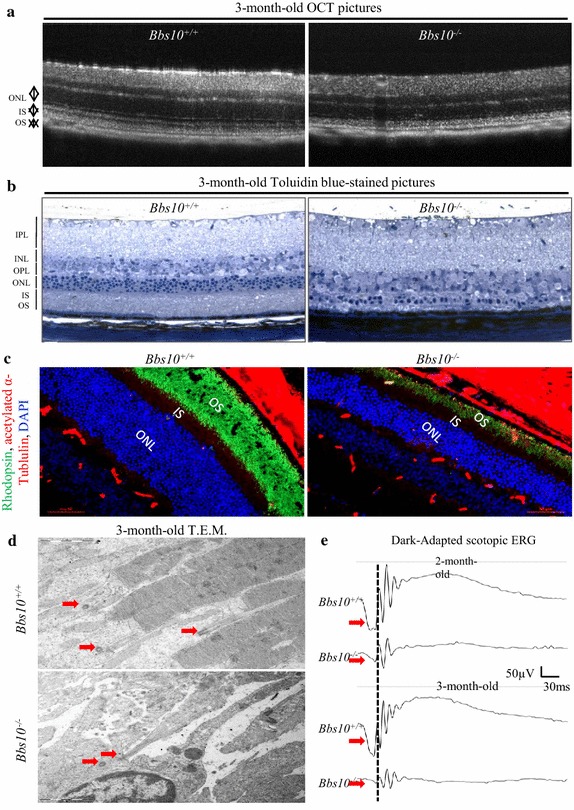
Retinal phenotype in *Bbs10* ^−*/*−^ mice. **a** Optical coherence tomography (OCT) pictures of retinas from *Bbs10* ^+*/*+^ and *Bbs10* ^−*/*−^ mice. (*OS* outer segment, *IS* inner segment, *ONL* outer nuclear layer) (n = 3 mice per genotype). **b** Toluidine blue-stained pictures of 7-µm-thick retinal sections from *Bbs10* ^+*/*+^ and *Bbs10* ^−*/*−^ mice (*OS* outer segment, *IS* inner segment, *ONL* outer nuclear layer, *OPL* outer plexiform layer, *INL* inner nuclear layer, *IPL* inner plexiform layer) *Scale bar* 20 µm. **c** Immunofluorescence of 7-µm-thick retinal sections from *Bbs10* ^+*/*+^ and *Bbs10* ^−*/*−^ mice. Nuclei were counterstained with DAPI (*blue*), rhodopsin (*green*), acetylated a-tubulin (*red*). **d** Transmission electron microscopy (T.E.M.) pictures of retinal sections in *Bbs10* ^+*/*+^ and *Bbs10* ^−*/*−^ mice (*ROS* retinal outer segment, *RIS* retinal inner segment, *RPE* retinal pigment epithelium). *Red arrows* indicate the centrioles and the connecting cilium. **e** Scotopic electroretinograms (ERG) recordings of 2- and 3-month-old *Bbs10* ^+*/*+^ and *Bbs10* ^−*/*−^ mice. *Red arrows* indicate a-waves on the ERG recordings (n = 6–8 per genotype).

*Bbs10*^−*/*−^ mice, like all reported BBS mouse models [3], exhibit severe retinal degeneration. 3-month-old *Bbs10*^−*/*−^ mice showed retinal thinning observed in the Optical Coherence Tomography (OCT) (Fig. 3a) and on histological toluidine-stained sections (Fig. 3b). Immunostained cryosections of the corresponding retinas (Fig. 3c) for rhodopsin showed correct localisation in the outer segment (in green) albeit a markedly clear reduction of rhodopsin content in the *Bbs10*^−*/*−^ retinas. Transmission electron microscopy (T.E.M.) analysis revealed that the retinal phenotype was primarily due to the loss of the inner and outer segment (IS/OS) of the photoreceptors and the outer nuclear layer (ONL); although the connecting cilium could still be detected, next to the centriole (Fig. 3d, red arrows). This gradual loss of the photoreceptors in the *Bbs10*^−*/*−^ retinas correlated with a significant decrease of the a-wave (Fig. 3e, red arrows) and b-wave magnitudes of the scotopic electroretinograms (ERGs) in the *Bbs10*^−*/*−^ mice. We also found in the retinas of Bbs10^−/−^ mice an elevated number of TUNEL-positive nuclei indicating an ongoing process of apoptosis in the *Bbs10*^−*/*−^ retinas (Additional file 4: Figure S3A, B).

### Structural renal abnormalities associated with functional impairment are observed in *Bbs10*^−*/*−^ mice

**Fig. 4:**
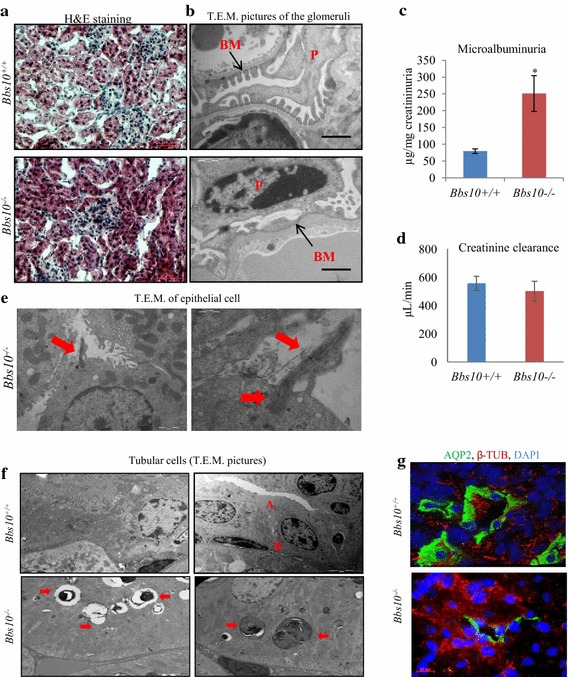
Renal phenotype of the *Bbs10* ^−*/*−^ mice and *Bbs10* ^*fl/fl*^ *; Cadh16*-*Cre* ^+*/*−^ mice. **a** Pictures of hematoxylin and eosin-stained kidney sections of 3-month-old *Bbs10* ^+*/*+^ and *Bbs10* ^−/−^ mice. **b** Transmission electron microscopy picture of kidney sections of the glomerular region from *Bbs10* ^+*/*+^and *Bbs10* ^−*/*−^ mice (*P* podocyte, *BM* basement membrane). *Scale bars* 5 and 2 µm. **c** Microalbuminuria levels from *Bbs10* ^+*/*+^ and *Bbs10* ^−/−^ mice. **d** Creatinine clearance levels (µL/min) and urinary volume under fluid deprivation of *Bbs10* ^+*/*+^ and *Bbs10* ^−/−^ mice (n = 7–9). Values are expressed as mean ± SEM. **e** Transmission electron microscopy picture of kidney epithelial cells from *Bbs10* ^+*/*+^, *Bbs10* ^−*/*−^ mice. *Red arrows* indicate the centrioles and the connecting cilium. **f** Transmission electron microscopy picture of kidney tubular cells from *Bbs10* ^+*/*+^, *Bbs10* ^−*/*−^ mice. *Red arrows* indicate cytoplasmic vacuoles, *A* apical side, *B* Basolateral side. **g** Pictures of immunostained kidney sections for AQP2 (*green*) and β-tubulin (*red*) from *Bbs10* ^+*/*+^and *Bbs10* ^−/−^ mice. Nuclei were counterstained with DAPI (*blue*). *Scale bars* 20 µm. Values are expressed as mean ± SEM. *p < 0.05

We next investigated the impact of *Bbs10* inactivation on the kidneys. In the glomerular region, T.E.M. analysis revealed substantial decrease of the glomerular basement membrane thickness combined with an absence of primary and secondary podocyte structures (Fig. 4a, b). This structural defect correlated with significant increase in albuminuria (Fig. 4c) although creatinine clearance was maintained in the *Bbs10*^−*/*−^ mice (Fig. 4d). As it was reported that BBS protein inactivation could impair ciliogenesis [14, 20, 21], this prompted us to verify the ciliated status of the tubular epithelial cells. Ciliated epithelial cells were readily detected in the *Bbs10*^−*/*−^ tubular epithelium as depicted by T.E.M analysis (Fig. 4e). Further analysis showed correctly polarized epithelial cells for both proximal tubular and distal convoluted epithelial cells in the *Bbs10*^−*/*−^ kidneys but revealed large intracytoplasmic inclusions in the *Bbs10*^−*/*−^ epithelial cells (Fig. 4f and Additional file 5: Figure S4). No detectable cystic lesion were detected and correct targeting of the Aquaporin 2 (AQP2) to the apical side of the epithelial cells was found in the *Bbs10*^−*/*−^ epithelial cells (Fig. 4g).

### *Bbs10* ^*fl/fl*^*; Cadh16Cre*^+*/*−^ exhibit no detectable impairment in renal morphology or function

**Fig. 5:**
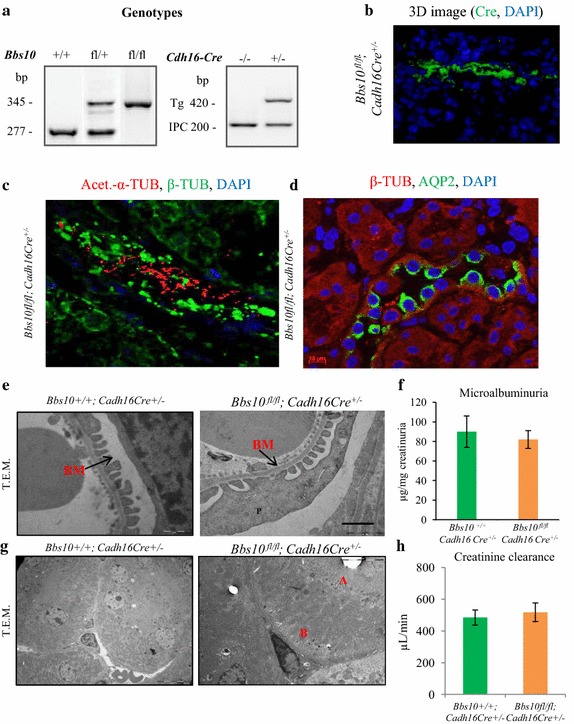
Renal phenotype of the *Bbs10* ^*fl/fl*^ *; Cadh16*-*Cre* ^+*/*−^ mice. **a** PCR genotyping of heterozygous WT (*Bbs10* ^+*/*+^) (*Bbs10* ^*fl/*+^), floxed (*Bbs10* ^*fl/fl*^) mice non-*Cre* (*Cre* ^−*/*−^) and heterozygous (*Cre* ^+*/*−^) genotyping (*bp* base pairs). **b** 3D image of a kidney section immunostained for Cre (*green*) and nuclei counterstained with DAPI (*blue*). **c** 3D image of a kidney section immunostained for the primary cilia (acetylated α-tubulin in *red*), cytoskeleton (β-tubulin in *green*) and nuclei counterstained with DAPI (in *blue*). **d** Pictures of immunostained kidney sections for AQP2 (*green*) and β-tubulin (*red*) from *Bbs10* ^*fl/fl*^ *; Cadh16Cre* ^+*/*−^ mice. Nuclei were counterstained with DAPI (*blue*). *Scale bars* 20 µm. **e** T.E.M. analysis of epithelial cells in the proximal tubule from *Bbs10* ^+*/*+^ *; Cadh16Cre* ^+*/*−^and *Bbs10* ^*fl/fl*^ *; Cadh16Cre* ^+*/*−^ mice. *BM* basement membrane. **f** Microalbuminuria levels for of *Bbs10* ^*fl/fl*^ *; Cadh16Cre* ^+*/*−^ and control *Bbs10* ^+*/*+^ *; Cadh16Cre* ^+*/*−^ littermates (n = 6–7 per genotype). **g** T.E.M. analysis of epithelial cells in the distal tubule from *Bbs10* ^+*/*+^ *; Cadh16Cre* ^+*/*−^ and *Bbs10* ^*fl/fl*^ *; Cadh16Cre* ^+*/*−^ mice. **h** Creatinine clearance levels (µL/min) and urinary volume under fluid deprivation of *Bbs10* ^*fl/fl*^ *; Cadh16Cre* ^+*/*−^ and control *Bbs10* ^+*/*+^ *; Cadh16Cre* ^+*/*−^ littermates (n = 7–9). Values are expressed as mean ± SEM. *p < 0.05.

Next we generated *Bbs10* ^*fl/fl*^*; Cadh16Cre*^+*/*−^ and their controls *Bbs10*^+*/*+^*; Cadh16Cre*^+*/*−^ and genotyped those by PCR approach (Fig. 5a). The *Bbs10* ^*fl/fl*^*; Cadh16Cre*^+*/*−^ displayed Cre expression in the renal epithelium (Additional file 3: Figure S2A) and the Cre recombinase activity was validated by detecting green fluorescence signal in the renal epithelial cells of the *Cadh16Cre*^+*/*+^*; RosaTomatoeGFP*^+*/*+^ (Additional file 3: Figure S2B). To further establish specific Cre-mediated excision of *Bbs10* in the renal epithelial cells, we performed an immunodetection of BBS10 in the tubular renal epithelial cells and found no cells positively stained for BBS10 in the *Bbs10* ^*fl/fl*^*; Cadh16Cre*^+*/*−^ compared to control (Additional file 6: Figures S6A, B). As *Cadh16*-*Cre* expression targets specifically the renal epithelial cells, no other characteristic BBS phenotype like obesity or retinal degeneration was observed in the *Bbs10* ^*fl/fl*^*; Cadh16Cre*^+*/*−^ mice (data not shown). Immunostaining of acetylated-α-tubulin on kidney cryosections showed the presence of ciliated cells in *Bbs10* ^*fl/fl*^*; Cadh16Cre*^+*/*−^ mice (Fig. 5c) together with normal apical targeting of AQP2 in the epithelial cells in the *Bbs10* ^*fl/fl*^*; Cadh16Cre*^+*/*−^ mice (Fig. 5d). These data demonstrate the correct polarization of the BBS10-deprived renal epithelial cells. Moreover, since we did not target the glomerular region with the *Cadh16Cre*, no structural defect was observed in any of the podocyte structures of the *Bbs10* ^*fl/fl*^*; Cadh16Cre*^+*/*−^ mice (Fig. 5e), an effect correlated with normal microalbuminuria levels (Fig. 5f). Surprisingly, no cytoplasmic inclusion was observed in the epithelial cells in both the proximal and distal tubular regions (Fig. 5g) and creatinine clearance was comparable between the tested groups (Fig. 5h). These results indicate that the sole *Bbs10* inactivation in the renal epithelial cells is not enough to trigger massive pathological stress in the renal epithelium.

### Systemic Bbs10 inactivation is a prerequisite for the classical polyuria phenotype in vivo

**Fig. 6:**
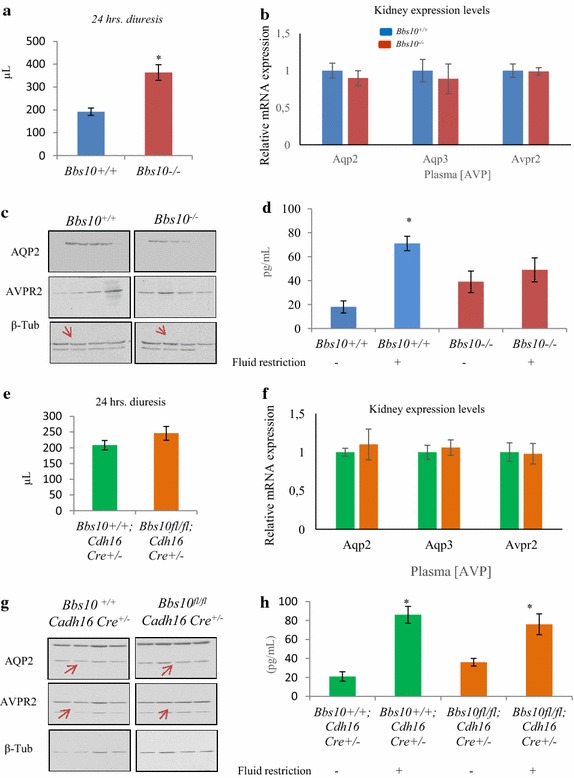
Bbs10^−/−^ mice but not *Bbs10* ^*fl/fl*^ *; Cadh16*-*Cre* ^+*/*−^ mice display polyuria associated with increased circulating AVP concentrations. **a** Urinary volume (µL) under 24-h fluid deprivation of *Bbs10* ^+*/*+^ and *Bbs10* ^−/−^ mice (n = 6 per genotype). **b** Relative mRNA expression levels of *Aqp2*, *Aqp3* and *Avpr2* in *Bbs10* ^+*/*+^ and *Bbs10* ^−/−^ kidneys. Gene of reference *Gapdh*. **c** Entire photographs of immunoblots for AQP2, AVPR2 and β-tub in kidneys of *Bbs10* ^+*/*+^ and *Bbs10* ^−*/*−^. **d** AVP plasma levels in *Bbs10* ^+*/*+^ and *Bbs10* ^−*/*−^ under 24-h fluid restriction or in normal conditions. (n = 5 per genotype). **e** Urinary volume (µL) under 24-h fluid deprivation of *Bbs10* ^*fl/fl*^ *; Cadh16Cre* ^+*/*−^ and control *Bbs10* ^+*/*+^ *; Cadh16Cre* ^+*/*−^ littermates (n = 6 per genotype). **f** Relative mRNA expression levels of *Aqp2*, *Aqp3* and *Avpr2* in *Bbs10* ^+*/*+^ *; Cadh16Cre* ^+*/*−^ and *Bbs10* ^*fl/fl*^ *; Cadh16Cre* ^+*/*−^. **g** Entire photographs of the immunoblots for AQP2, AVPR2 and β-tubulin (β-tub) in kidneys of *Bbs10* ^+*/*+^ *; Cadh16Cre* ^+*/*−^ and *Bbs10* ^*fl/fl*^ *; Cadh16Cre* ^+*/*−^; the immunoblots are shown in Additional file 4: Figure S4A, B. **h** AVP plasma levels in *Bbs10* ^*fl/fl*^ *; Cadh16Cre* ^+*/*−^ and control *Bbs10* ^+*/*+^ *; Cadh16Cre* ^+*/*−^ under 24-h fluid restriction or in normal conditions. (n = 5 per genotype). Values are expressed as mean ± SEM. *p < 0.05.

The most prominent renal phenotype in BBS patients is polyuria; a phenotype which was linked to a defective AVP signalling cascade. To identify the cellular mechanism behind this renal phenotype, we tested the *Bbs10*^−*/*−^ mice for fluid retention. The mice underwent 24-h fluid deprivation and the collected urinary volumes were analysed. *Bbs10*^−*/*−^ mice suffered of increased diuresis (Fig. 6a). This increased discharge of urine correlated with a decrease in mRNA expression of aquaporin (*Aqp2*), aquaporin 3 (*Aqp3*) and *Avpr2* (Fig. 6b), nor decrease in protein levels of AQP2 and AVPR2 (Fig. 6c). Circulating AVP levels revealed a similar drastic increase in the *Bbs10*^−*/*−^ mice irrespective of fluid intake (Fig. 6d), contrasting with the *Bbs10*^+*/*+^ mice that exhibit the normal physiological response upon the fluid restriction. Next, we performed the same series of tests with the *Bbs10* ^*fl/fl*^*; Cadh16Cre*^+*/*−^ mice, to verify whether the specific *Bbs10* inactivation could impair AVP signalling in the kidneys. Interestingly, neither the 24-h urinary volumes (Fig. 6e), nor renal mRNA expression levels of AQP2, AQP3 and AVPR2 (Fig. 6f) nor protein expression levels of AQP2 and AVPR2 (Fig. 6g) were impacted in the *Bbs10* ^*fl/fl*^*; Cadh16*-*Cre*^+*/*−^ mice. Compared to controls, normal physiological AVP response to fluid restriction was observed in the *Bbs10* ^*fl/fl*^*; Cadh16*-*Cre*^+*/*−^ mice (Fig. 6h). All together these data demonstrate that the specific inactivation of Bbs10 in the renal epithelial cells does not induce polyuria and the associated high AVP circulating levels.

## Discussion

Herein, we describe and compare the phenotypes of a *Bbs10*^−*/*−^ versus *Bbs10* ^*fl/fl*^*; Cadh16Cre*^+*/*−^ mice. The *Bbs10*^−*/*−^ mice recapitulated most clinical features of the human condition, ranging from early-onset obesity, retinal degeneration and renal dysfunction whereas the *Bbs10* ^*fl/fl*^*; Cadh16Cre*^+*/*−^ mice show neither detectable renal defect at 3 months nor any other noticeable manifestations. The *Cadh16 Cre* mouse used in this work to mediate the specific inactivation of *Bbs10* in renal epithelial cells was the Cre Ksp1.3/Cre transgenic mouse. This model carries the 1,329 bp fragments of the 5′ flanking region of the Ksp-cadherin promoter [19]. Two Cadh16-Cre mice were developed by the group of Igarashi in 2002 containing different fragments of the 5′ flanking region of the Ksp-cadherin promoter [19], one of the mice carrying the 1,329 bp (Ksp1.3/Cre mouse) and the other carrying the 324 bp fragment (Ksp0.3/Cre mice). Cre expression in Ksp1.3 mice was restricted to the tubular epithelial cells in the mature and developing kidney, while the Ksp0.3 mice produce a variegated expression pattern [19, 22]. In this work we used the Ksp1.3/Cre transgenic mice to specifically target the tubular epithelial cells of the kidney and no other renal cells types. Based on the results presented here and combined with the use of the specified Cre mouse line, it seems that specific *Bbs10* inactivation in the renal epithelium on its own, may not be sufficient to be deleterious to renal function up to 3 months. This being said, we cannot entirely exclude especially in absence of a phenotype in the *Bbs10* ^*fl/fl*^*; Cadh16Cre*^+*/*−^ that this lack of impact on the renal physiology could be due to a heterogeneous and variable activity of the Cre in vivo; a proof that will require further investigations.

On the other hand *Bbs10*^−*/*−^ mice developed progressive obesity associated with hyperphagia and hyperleptinemia. The absence of hypertrophic adipose tissue at 3 months in both *Bbs10*^−*/*−^ and *Bbs12*^−*/*−^ mice does not support the classical leptin resistance theory leading to obesity in the BBS (32). In fact, our results follow the same line of thought that was suggested by another group studying the *Bbs4*^−*/*−^ mice. The authors demonstrated that leptin resistance was not a constitutive defect but seemingly an acquired one appearing as the mice were ageing [13]. Besides, we and others have also shown that the inactivation of *BBS* genes was favouring pre-adipocyte lineage [14, 23] and adipogenesis [24]. These effects could explain the observed hyperplastic adipose tissue as well as the higher circulating levels of leptin observed in *Bbs10*^−*/*−^ mice (Fig. 2d); a phenotype observed in all BBS mouse models and in human patients (15, 16, 31, 30). Moreover, the 3-month-old *Bbs10*^−*/*−^ mice were not hyperglycaemic but exhibited a significant delay in bringing their glucose levels back to normal following GTT (Fig. 2f). Simultaneously, these mice showed insulin sensitivity, indicating that the delay in glucose handling observed with the GTT was not related to insulin resistance. Interestingly, it has been described that leptin could be as effective as insulin in controlling glucose levels [25]. It is, therefore, conceivable that the delay in glucose handling observed in the *Bbs10*^−*/*−^ mice could be due to the hyperleptinemia; a hypothesis that warrants further investigations with targeted tissue BBS inactivation. In this present study, 3-month-old mice were used to clearly demonstrate that in absence of obesity *Bbs10* inactivation has no major impact on renal function. Although it might seem interesting to have older mice to investigate the effect of *Bbs10* inactivation at later stages, it then becomes difficult to dissect the exact contribution of *Bbs* inactivation due to the effects of ageing itself.

Coincidently, the *Bbs10*^−*/*−^ mice suffered from RP. The retinal degeneration observed in the *Bbs10*^−*/*−^ was similar to the other previous *Bbs* mouse models (33, 34, 29, 27, 19), characterized by the loss of photoreceptors resulting in a thinning of the retina and a progressive flattening of the ERGs. Of note, is the presence of connecting cilium (Fig. 3c). With the complete photoreceptor structure, the *Bbs10*^−*/*−^ retina highlights the fact that BBS10, as was the case for BBS12 [26], is not required for ciliogenesis. The observed defect is probably related to an impaired ICT from the inner segment to the outer segment.

BBS renal phenotype is a mix of different defects of the nephron correlating with the variable human renal phenotype. We, therefore, investigated the effect of a total knockout condition versus a specific renal epithelial inactivation. In the *Bbs10*^−*/*−^ mice, the glomeruli suffered from the absence of the primary and secondary podocyte structures (Fig. 4b) correlating with elevated albuminuria (Fig. 4c). Accordingly, no such glomerulopathy was present in the *Bbs10* ^*fl/fl*^*; Cadh16Cre*^+*/*−^ mice (Fig. 5f, g), as the Cre activity was limited to the renal epithelial regions (Figure S2B). The primary cilium is known to play a role in development of the podocytes and that later in life, the podocytes lose their ciliated status as the flow gets stronger to prevent excessive polycystin-mediated calcium signalling [27]. As the observed structural defects in the *Bbs10*-deprived podocytes seemingly bear a developmental aspect and not an acquired one, we hypothesized that BBS10 is crucial for proper podocyte development. Surprisingly, human BBS patients do not present overt early-onset glomerulopathy but do exhibit decreasing glomerular function with age [28, 29]. This discrepancy could result from inherent differences between species as exemplified by the fact that rodent podocytes seem to be ciliated only during development [27] whereas human podocytes remain ciliated in fully differentiated and functional podocytes [30, 31].

Moreover, cyst was undetectable in both *Bbs10*^−*/*−^ and the *Bbs10* ^*fl/fl*^*; Cadh16Cre*^+*/*−^ kidneys (Figs. 4b–f, 5f–h and Additional file 5: Figure S4). This feature shared with other BBS chaperone KO mice, namely the *Bbs12*^−*/*−^, suggests that chaperone BBS inactivation does not impact the planar cell polarization (PCP) of the developing kidney like other ciliopathies. In addition, proper PCP of the *Bbs10*-deprived renal epithelial cells was further evidenced by the presence of primary cilia on the apical side of the epithelium (Figs. 4e, 5d) combined with the AQP2 apical localisation (Figs. 4h, 5e). Interestingly, only *Bbs2*^−*/*−^ and *Bbs4*^−*/*−^ mice suffered from late-onset cystogenesis which was secondary and uniquely associated to obesity [32]. Altogether, these data highlight that cyst formation is not, per se, intrinsically correlated to BBS gene inactivation but could nevertheless favour cystogenesis when combined with BBS-induced obesity. Besides cytoplasmic vacuoles, an established parameter for cellular stress of the renal epithelium [33] was observed in the *Bbs10*^−*/*−^ renal epithelial cells but not in the *Bbs10* ^*fl/fl*^*; Cadh16Cre*^+*/*−^ ones (Figs. 4g, 5h). These highlight the prerequisite of multiple defects to induce detectable renal defects.

Finally, polyuria, the most prominent human renal phenotype in BBS was observed in the *Bbs10*^−*/*−^ and not in *Bbs10* ^*fl/fl*^*; Cadh16Cre*^+*/*−^ mice. Given that AVPR2 is localized in the primary cilium of renal epithelial cells and that BBS protein inactivation in renal cells impairs their capacity to respond to AVP and activate their luminal AQP2 in vitro [15], we measured the protein levels of AQP2, AQP3 and AVPR2. No difference in expression levels of these proteins were found in both tested models. This indicates that polyuria in the *Bbs10*^−*/*−^ mice is not linked to a local decrease in expression levels of these proteins (Fig. 6b, c, f, g). The fact that the AVP levels were always high in the *Bbs10*^−*/*−^ mice, irrespective of the fluid intake condition (Fig. 6d), highlights an AVP-resistant status in *Bbs10*^−*/*−^ mice; an effect which was absent in the *Bbs10* ^*fl/fl*^*; Cadh16Cre*^+*/*−^ mice (Fig. 6h). Based on these findings, we hypothesized that the BBS-induced polyuria is not simply related to the absence of BBS10 in the renal epithelium but is the result of more complex interactions between several pathways being impacted in the *Bbs10*^−*/*−^. These could be linked to the hypothalamic–pituitary axis sensing osmolality and secreting AVP compared to the other ciliated cells.

## Conclusions

The data presented herein describe the phenotype of a new mouse model for one of the most commonly mutated genes in BBS human patients, namely BBS10, with special emphasis in the kidney phenotype. Our findings show that deletion of *Bbs10* is able to recapitulate most of the clinical BBS features, whereas the *Bbs10* ^*fl/fl*^*; Cadh16Cre*^+*/*−^ did not induce any detectable defect. Overall, these results highlight once more the complexity of renal dysfunction characterizing this emblematic ciliopathy and like for the study of the origins of obesity, an integrative approach is required to understand and ultimately cure these genetic disorders.

## Additional files

Additional file 1: Supporting information: Methods; Additional Table 1: list of primers used for RT-qPCR; Additional Table 2: List of antibodies used. Supplementary references. Additional file 2:
**Figure S1.** Schematic representation of the DNA construct for the Knock out Allele. Additional file 3:
**Figure S2.** *Bbs10* ^*fl/fl*^ *; Cadh16Cre* ^+*/*−^ mice model. (A) Immunostaining of Cre recombinase on kidney sections of *WT* and *Bbs10* ^*fl/fl*^ *; Cadh16Cre* ^+*/*−^ mice counterstained with DAPI. (B) Fluorescence images of kidney sections from *RosaTomatoeGFP* ^+*/*−^ *; Cadh16Cre* ^+*/*−^ mice. Cells expressing *Cadh16*-Cre lineage marker GFP (green) are indicated with arrows. Scale bars: 200 μm (C) Growth curve of *Bbs10* ^*fl/fl*^ *; Cadh16Cre* ^+*/*−^ and control littermates (n = 8, mean ± SEM). (D) Relative mRNA expression of *Bbs10* gene in kidneys of *Bbs10* ^*fl/fl*^ and *Bbs10* ^+*/*+^. Reference gene: *Gapdh* (n = 4, mean ± SEM). Additional file 4:
**Figure S3.** Apoptosis of photoreceptors in *Bbs10* ^−*/*−^ mice. Immunofluorescence of 7 µm thick retinal sections from *Bbs10* ^+*/*+^ and *Bbs10* ^−*/*−^ mice. Fluorescence signal in green correspond to TUNEL signal and in blue the nuclei counterstained with DAPI. Additional file 5:
**Figure S4.** Transmission Electron Microscopy of kidney tubular cells from *Bbs10* ^+*/*^, *Bbs10* ^−*/*−^ and *Bbs10* ^*fl/fl*^ *; Cadh16Cre* ^+*/*−^ mice. Scale bars: 5 µm and 2 µm. Additional file 6:
**Figure S5.** Immunodetection of BBS10 in the *Bbs10* ^*fl/fl*^ *; Cadh16*-*Cre* ^+*/*−^ tubular region. (A) Representative epifluorescence picture of BBS10-immunostained cryosection of a distal tubule in the medullary region of mice with the indicated genotype. (B) 3D images of immunostained distal tubules against BBS10 for the indicated genotype from 3-month-old male mice generated from a Z-stack of epifluorescence pictures including the image showed in (A).

## Abbreviations

BBS: Bardet–Biedl syndrome
BBS10: Bardet–Biedl syndrome gene 10
KO: knock out
ERGs: scotopic electroretinograms
GTT: glucose tolerance test
ICT: intraciliary transport
IS: inner segment
ITT: insulin tolerance test
ONL: outer nuclear layer
OS: outer segment
RP: retinitis pigmentosa
T.E.M: transmission electron microscopy
AQP2: aquaporin 2
AQP3: aquaporin 3
AVP: arginine vasopressin
AVPR2: arginine, vasopressin receptor 2
