# Supplementary material for: Comparing the Bbs10 complete knockout phenotype with a specific renal epithelial knockout one highlights the link between renal defects and systemic inactivation in mice
Source: Cilia. 2015 Aug 13;4:10. doi: 10.1186/s13630-015-0019-8 (PMC4535764; doi:10.1186/s13630-015-0019-8)
Supplement: Additional file 1: — Supporting information: Methods; Additional Table 1: list of primers used for RT-qPCR; Additional Table 2: List of antibodies used. Supplementary references. [file 13630_2015_19_MOESM1_ESM.pdf]

Supporting Information

Comparing the *Bbs10* complete knockout phenotype with a specific renal epithelial knockout one highlights the link between renal defects and systemic inactivation in mice

Noëlle Cognard, Maria J Scerbo, Cathy Obringer, Xiangxiang Yu, Fanny Costa, Elodie Haser, Dane Le, Corinne Stoetzel, Michel J Roux, Bruno Moulin, Hélène Dollfus, Vincent Marion

Methods

Fluid deprivation studies

Mice were fed ad libitum and had free access to water until 12 weeks and then they were transferred in Tecniplast® diuresis cage (Catalog: # 01-288-2D, Tecniplast®, West Chester, Pennsylvania, USA) and were undisturbed during 5 days for habituation. On the 6th day, water was removed for 24h and on the 7th day, mice were euthanized by decapitation. Urine was collected for diuresis measurements and plasma and organs were isolated and stored at -80°C for analysis

Histological studies and immunofluorescence experiments

Kidneys and eyes freshly sampled were included in Optimal Cutting Temperature Compound^{TM} (OCT^{TM}, Catalog #: 4583, Tissue-Tek® OCT^{TM}, Sakura® Finetek, Torrance, California, USA) and cryosections of 7µm were cut with Cryostat Leica CM1950 (Catalog #: 14 0477 8001, Leica Biosystems, Wetzlar, Germany). Kidney sections were treated following manufacturer's procedure for Haematoxylin - Eosin staining (Catalog #: HHS-16 Sigma-Aldrich Saint-Louis, Missouri, USA). Cryosections were fixated in 10% Formalin for 15 minutes (Catalog #: F555-4L, Sigma-Aldrich, Saint-Louis, Missouri, USA) and then permeabilized with 0.2% SDS-PBS for 30 seconds. Blocking solution used was 5%-Bovine Serum Albumin (BSA) in PBS. Primary antibodies were diluted in blocking solution and incubated overnight and secondary antibodies were diluted in PBS. Nuclei were counterstained with Hoechst (Catalog #: D1306, Invitrogen, Carlsbad, California, USA).

Slides were then mounted with Vectashield® Mounting Medium (Catalog #: H-1000, Vector Laboratories, Burlingame, California, USA). For BBS10 immunostaining, BBS10 antibody was preadsorbed in order to remove excessive unspecific background of the antibody. Briefly, the blocking solution containing the BBS10 antibody was incubated on a cryosection of a *Bbs10*$^{-/-}$ kidney for 1.5 hours at room temperature. The supernatant was then recovered and preadsorbed in a second incubation round on a new cryosection for another 1.5 hours at R.T. The supernatant of this second preadsorbtion was then used for the immunostaining. Images were acquired and analyzed with Zeiss Imager.Z2 microscope and driven by ZEN 2012 software (Carl Zeiss Inc., Oberkochen, Germany).

Electron Microscopic analysis

The samples were fixed by immersion in 2.5% Glutaraldehyde and 2.5% Paraformaldehyde in Cacodylate buffer (0.1M, pH 7.4), and post fixed in 1% osmium tetroxide in 0.1M Cacodylate buffer for 1 hour at 4°C and dehydrated through graded alcohol (50, 70, 90, 100%) and propylene oxide for 30 minutes each. Samples were embedded in Epon™ 812 (Sigma-Aldrich, Saint-Louis, Missouri, USA). Semi-thin sections were cut at 2µm with an ultra-microtome (Leica Ultracut UCT, Leica Biosystems, Wetzlar, Germany) and stained with toluidine blue, and histologically analyzed by light microscopy. Ultrathin sections were cut at 70nm and contrasted with uranyl acetate and lead citrate and examined at 70kv with a Morgagni 268D electron microscope. Images were captured digitally by Mega View III camera (Soft Imaging System).

Metabolic and biochemical studies

Mice were maintained on a 12h-light/12h-dark cycle and fed ad libitum normal chow diet (LM-485, Harlan Teklad Premier Laboratory Diets, Harlan Laboratories, Indianapolis, Indiana, USA). After weaning, body weight and food intake were determined. Animals were acclimatized to the procedure room and handling for injections. The mice were fasted

overnight prior to testing glyceamia. For the Glucose Tolerance Tests (GTT), mice were given an IP injection of glucose (2g/kg of body weight; 45% glucose). For Insulin Tolerance Tests (ITT) they were injected with 0.5 units/kg of insulin (Humalog® 100UI/ml Catalog #: A761338 Lilly). Blood was obtained from the tail vein at 0, 30, 60, 90, 120 min after glucose injection for GTT or at 0, 15, 30, 45, 60 min after insulin injection for ITT. Glucose levels were measured with an Optium Xceed glucose meter (Abbott Diabetes Care Ltd.). Circulating plasma levels of Leptin and Arginine-Vapsopressin (AVP), Creatinine as well as creatinuria and microabluminuria concentrations obtained after 24 hrs fluid deprivation were measured by ELISA (Catalog #: EZML-82K, Millipore, Billerica, Massachusetts, USA; #KA0301, Abnova, Taipei, Taiwan; Catalog #: 7000460 and Catalog #: 500701, Cayman Chemicals, Arbor, Michigan, USA and Catalog #: KT-343, Kamiya Biomedical Company, Thousand Oaks, California, USA respectively). Creatinine Clearance was estimated using the formula UxV/P (U: Urinary Creatinine, V: 24h-urine volume, P: Plasma Creatinine).

Visual phenotyping

Electroretinograms (ERGs) were performed with the HMsERG system (Ocuscience®, Kansas City, Missouri, USA). Mice were dark-adapted overnight and then anesthetized with IP injection of Domitor (7.6µg/g) (Domitor 1mg/ml, Janssen-Cilag, Issy-les-Moulineaux, France) and ketamine (760µg/g) (Ketamine 1000, Virbac, Carros, France). Pupils were dilated with Atropine eye drops (Atropine Alcon 0.3%, Alcon, Rueil-Malmaison, France). ERGs standard operating procedure was performed according to manufacturer’s protocol (Ocuscience®, Kansas City, Missouri, USA). Briefly, the protocol consisted in recording a dark-adapted ERG after photonic stimuli with intensities ranging from 0.1 to 25cd.s/m^2. ERG scotopic results were amplified and captured digitally by ERG View system 4.3 (Xenotec, Ocuscience®, Kansas City, Missouri, USA). Optical coherence tomography (OCT) was carried out on anesthetized mice using the Bioptigen SD800 apparatus.

Immunoblotting analysis

Kidneys protein extracts were obtained using RIPA buffer and lysates were sonicated using a sonicator (Omni Sonic Ruptor 250, Omni International™, Kennesaw, Georgia, USA). 80µg of total protein were loaded on 10% denaturing polyacrylamide-SDS gel. Then protein transfer was performed on nitrocellulose membrane (Catalog #: 9004-70-0, BioRad, Hercules, California, USA) and membranes were blocked with 5% milk in TBST (500mM Tris, pH 7.4, 1.5M NaCl, 0.05 % Tween). Primary and secondary antibodies were incubated in blocking solution. SuperSignal West Femto Maximum Sensitivity Substrate® (Catalog #: 34095, Thermo Scientific, Waltham, Massachusetts, USA) and Novex® ECL HRP Chemiluminescent Substrate Reagent Kit (Catalog #: WP20005, Invitrogen, Carlsbad, California, USA) were used for visualizing antibody binding. Images were acquired on the Molecular Imager® Versadoc™ MP4000 System (BioRad, USA).

RNA extraction and real-time PCR

RNA extraction on tissues was performed using TRIzol® reagent (Catalog #: 15596-018, Invitrogen®, Life Technologies™, Carlsbad, California, USA) and TissueRuptor® (Catalog #: 9001272, Qiagen, Venlo, Nederland). RNA samples were treated with TURBO™ DNA-free Kit (Catalog #: AM1907, Ambion®, Life Technologies™, Carlsbad, California, USA) prior to reverse transcription using the iScript® cDNA synthesis kit (Catalog #: 170-889, BioRad, USA). Real-Time qPCR was performed using the iQ SYBR® Green Supermix (Catalog #: 170-8886, BioRad, USA) on C1000™ thermocycler (CFX96, Real-Time System, BioRad, USA). Quantitative gene expression was calculated by the $\Delta\Delta C_t$ method relative to *Gapdh* as reference gene.

Additional table 1: list of primers used for RT-qPCR

**Genes**	**references**
Mu-Bbs10-Rt-F1	TGCTTAGCAGGGATGGAG
Mu-Bbs10-Rt-R1	TTGCAGAGCCTGGGAAATAG
Mu-Bbs10-Rt-F2	GGCCAAGTGTTGTGTACGAA
Mu-Bbs10-Rt-R2	GAATGAATCTTTTCCTTTTTCTCC
Aqp2	Mm_*Aqp2*_1_SG QT00113610
Avpr2	Mm_*Avpr2*_1_SG QT00315315
Aqp3	Mm_*Aqp3*_2_SG QT01540623
Gapdh	Mm_*Gapdh*_3_SG QT01658692

Additional table 2: Antibodies used in this study

Antibodies	Catalog #	Company
**AVPR2** rabbit polyclonal	67788	Sigma, USA
**AQP2** rabbit polyclonal	sc-28629	Santa Cruz, France
**AQP3** goat polyclonal	sc-9885	Santa Cruz, France
**BBS10** rabbit polyclonal	In house; 12421-2-AP	Described in [1] ProteinTech
**B-Tubulin** mouse monoclonal	1TUB-2A2	Euromedex, France
**Donkey anti-rabbit** FITC	sc-2090	Santa Cruz
**Donkey anti-goat** TR	sc-2783	Santa Cruz
**Chicken anti-rabbit** HRP	sc-2955	Santa Cruz
**Goat anti-mouse** HRP	sc-2060	Santa Cruz
**Donkey anti-goat** HRP	sc-3851	Santa Cruz

Supplementary references

1. Marion V, Stoetzel C, Schlicht D, Messaddeq N, Koch M, Flori E, Danse JM, Mandel JL, Dollfus H: **Transient ciliogenesis involving Bardet-Biedl syndrome proteins is a fundamental characteristic of adipogenic differentiation**. *Proceedings of the National Academy of Sciences of the United States of America* 2009, **106**(6):1820-1825.

Supplementary figure Legends

Additional Figure 1: Schematic representation for the targeting strategy to generate the *Bbs10*$^{fl/fl}$ mouse model

Additional Figure 2: Apoptosis of photoreceptors in *Bbs10*$^{-/-}$ mice.

Immunofluorescence of 7µm thick retinal sections from *Bbs10*$^{+/+}$ and *Bbs10*$^{-/-}$ mice. Fluorescence signal in green correspond to TUNEL signal and in blue the nuclei counterstained with DAPI.

Additional Figure 3: Transmission Electron Microscopy of kidney tubular cells from *Bbs10*$^{+/}$, *Bbs10*$^{-/-}$ and *Bbs10*$^{fl/fl}$; *Cadh16Cre*$^{+/-}$ mice

Scale bars: 5µm and 2 µm

Additional Figure 4: *Bbs10*$^{fl/fl}$; *Cadh16Cre*$^{+/-}$ mice model

(A) Immunostaining of Cre recombinase on kidney sections of *WT* and *Bbs10*$^{fl/fl}$; *Cadh16Cre*$^{+/-}$ mice counterstained with DAPI. (B) Fluorescence images of kidney sections from *RosaTomatoeGFP*$^{+/-}$; *Cadh16Cre*$^{+/-}$ mice. Cells expressing *Cadh16*-Cre lineage marker GFP (green) are indicated with arrows. Scale bars: 200 µm (C) Growth curve of *Bbs10*$^{fl/fl}$; *Cadh16Cre*$^{+/-}$ and control littermates (n = 8, mean ± SEM). (D) Relative mRNA expression of *Bbs10* gene in kidneys of *Bbs10*$^{fl/fl}$ and *Bbs10*$^{+/+}$. Reference gene: *Gapdh* (n=4, mean ± SEM).

Additional Figure 5: Immunodetection of BBS10 in the *Bbs10*$^{fl/fl}$*; Cadh16-Cre*$^{+/-}$ tubular region

(A) Representative epifluorescence picture of BBS10-immunostained cryosection of a distal tubule in the medullary region of mice with the indicated genotype.

(B) 3D images of immunostained distal tubules against BBS10 for the indicated genotype from 3-month-old male mice generated from a Z-stack of epifluorescence pictures including the image showed in (A).
