# Supplementary material for: Comparing the Bbs10 complete knockout phenotype with a specific renal epithelial knockout one highlights the link between renal defects and systemic inactivation in mice
Source: Cilia. 2015 Aug 13;4:10. doi: 10.1186/s13630-015-0019-8 (PMC4535764; doi:10.1186/s13630-015-0019-8)
Supplement: Additional file 2: — Figure S1. Schematic representation of the DNA construct for the Knock out Allele. [file 13630_2015_19_MOESM2_ESM.pdf]

Schematic representation of the DNA construct for the Knock out allele

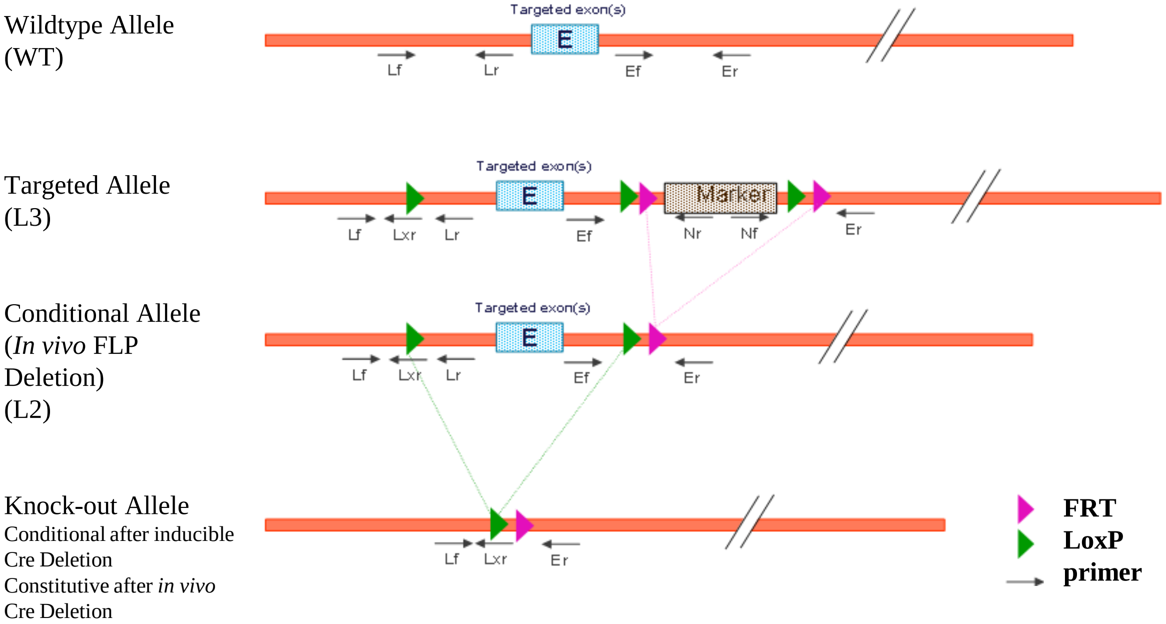

Additional Figure 1
