# Supplementary figures and images for: Comparing the Bbs10 complete knockout phenotype with a specific renal epithelial knockout one highlights the link between renal defects and systemic inactivation in mice
Source: Cilia. 2015 Aug 13;4:10. doi: 10.1186/s13630-015-0019-8 (PMC4535764; doi:10.1186/s13630-015-0019-8)

# Cre recombinase expression

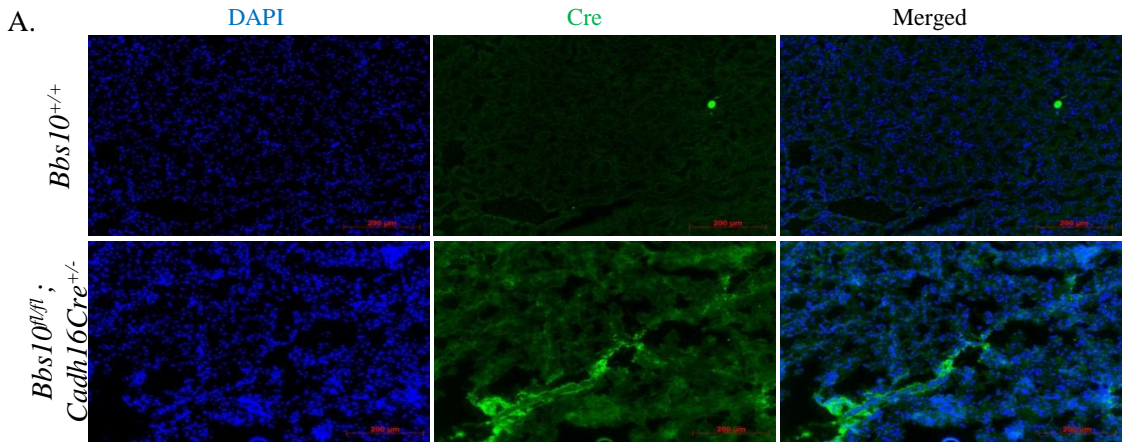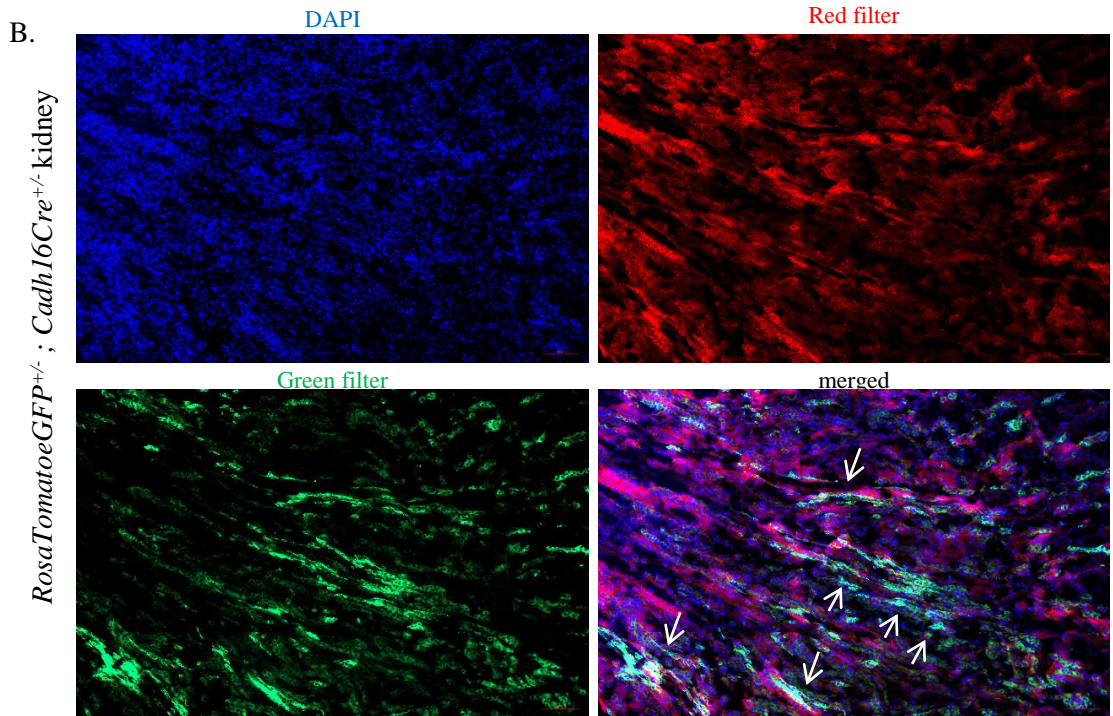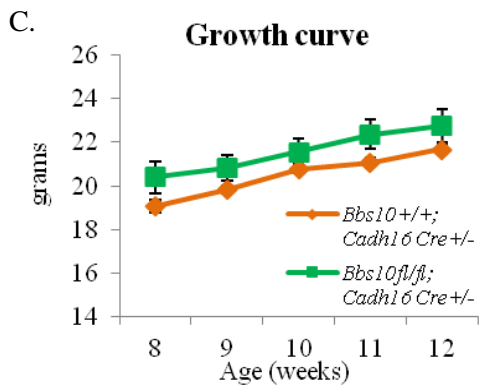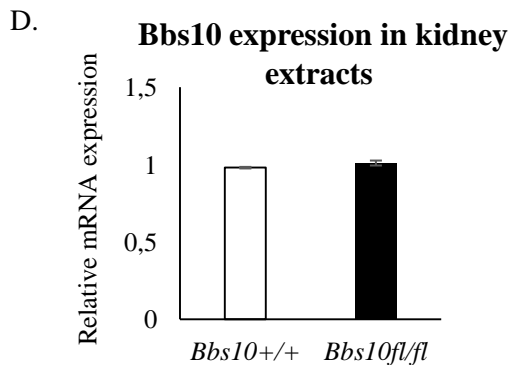

Supplement: Additional file 3: — Figure S2. Bbs10 fl/fl ; Cadh16Cre +/− mice model. (A) Immunostaining of Cre recombinase on kidney sections of WT and Bbs10 fl/fl ; Cadh16Cre +/− mice counterstained with DAPI. (B) Fluorescence images of kidney sections from RosaTomatoeGFP +/− ; Cadh16Cre +/− mice. Cells expressing Cadh16-Cre lineage marker GFP (green) are indicated with arrows. Scale bars: 200 μm (C) Growth curve of Bbs10 fl/fl ; Cadh16Cre +/− and control littermates (n = 8, mean ± SEM). (D) Relative mRNA expression of Bbs10 gene in kidneys of Bbs10 fl/fl and Bbs10 +/+. Reference gene: Gapdh (n = 4, mean ± SEM). [file 13630_2015_19_MOESM3_ESM.pdf]
