# Supplementary material for: Comparing the Bbs10 complete knockout phenotype with a specific renal epithelial knockout one highlights the link between renal defects and systemic inactivation in mice
Source: Cilia. 2015 Aug 13;4:10. doi: 10.1186/s13630-015-0019-8 (PMC4535764; doi:10.1186/s13630-015-0019-8)
Supplement: Additional file 4: — Figure S3. Apoptosis of photoreceptors in Bbs10 −/− mice. Immunofluorescence of 7 µm thick retinal sections from Bbs10 +/+ and Bbs10 −/− mice. Fluorescence signal in green correspond to TUNEL signal and in blue the nuclei counterstained with DAPI. [file 13630_2015_19_MOESM4_ESM.pdf]

Apoptosis of photoreceptors in Bbs10 total KO

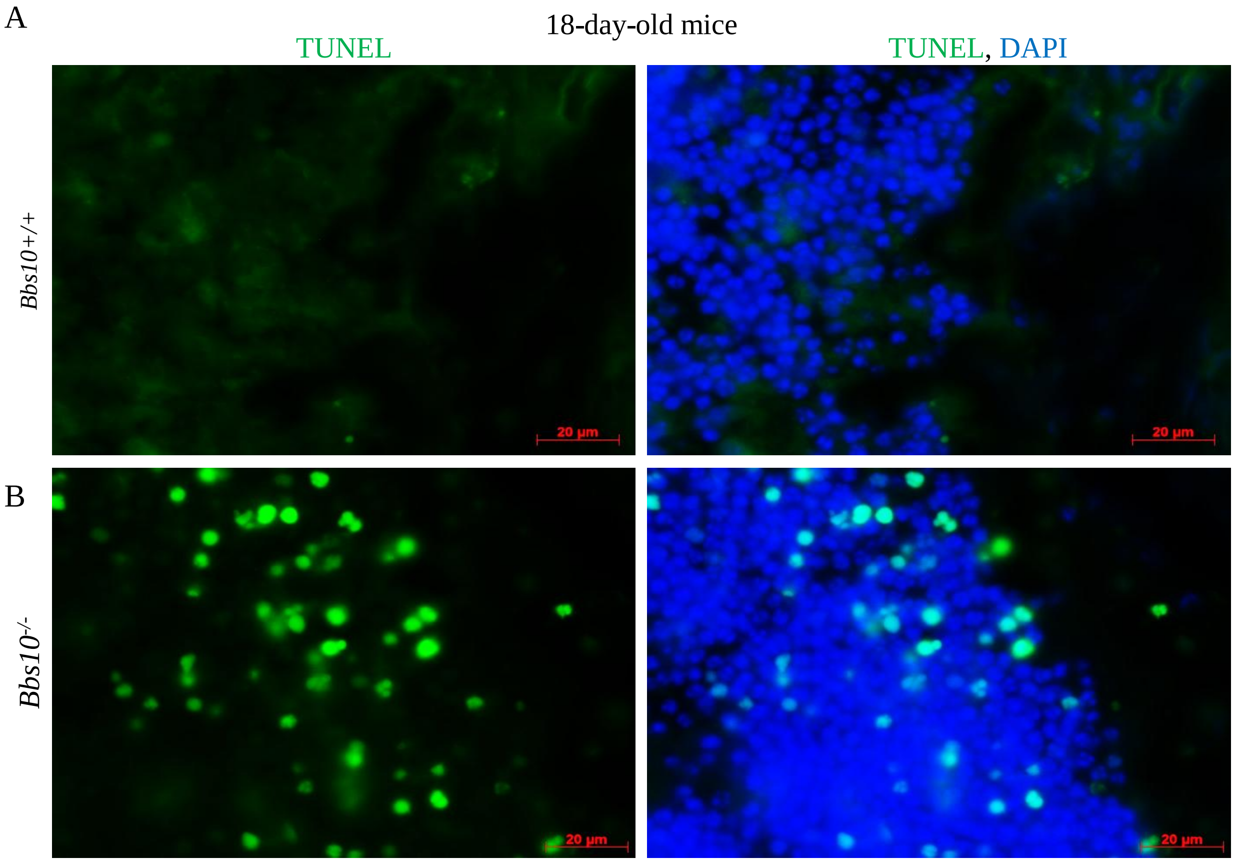

Additional Figure 3
