# Supplementary material for: Comparing the Bbs10 complete knockout phenotype with a specific renal epithelial knockout one highlights the link between renal defects and systemic inactivation in mice
Source: Cilia. 2015 Aug 13;4:10. doi: 10.1186/s13630-015-0019-8 (PMC4535764; doi:10.1186/s13630-015-0019-8)
Supplement: Additional file 5: — Figure S4. Transmission Electron Microscopy of kidney tubular cells from Bbs10 +/, Bbs10 −/− and Bbs10 fl/fl ; Cadh16Cre +/− mice. Scale bars: 5 µm and 2 µm. [file 13630_2015_19_MOESM5_ESM.pdf]

Transmission Electron Microscopy of kidney tubular cells

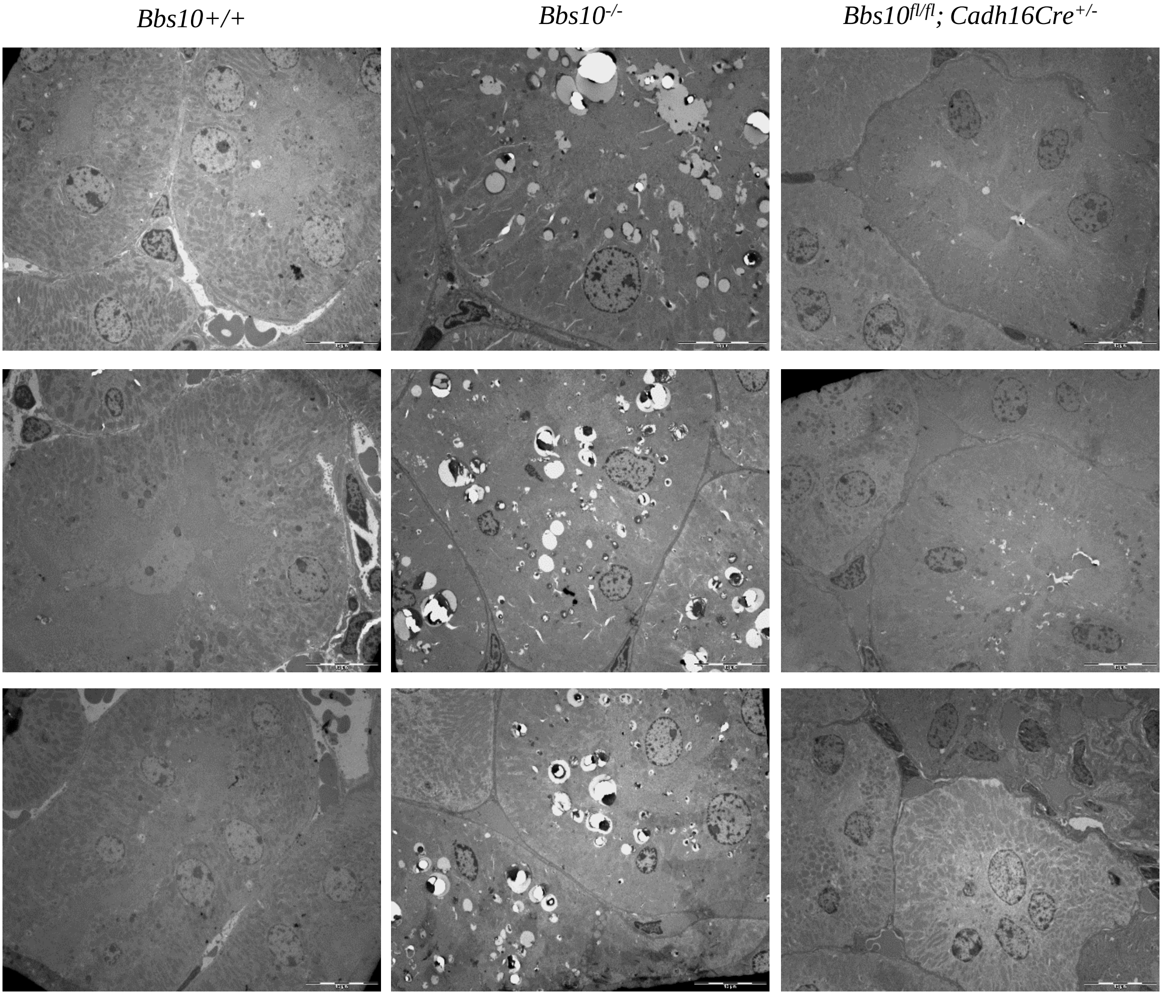

Additional Figure 4
