# Supplementary material for: Comparing the Bbs10 complete knockout phenotype with a specific renal epithelial knockout one highlights the link between renal defects and systemic inactivation in mice
Source: Cilia. 2015 Aug 13;4:10. doi: 10.1186/s13630-015-0019-8 (PMC4535764; doi:10.1186/s13630-015-0019-8)
Supplement: Additional file 6: — Figure S5. Immunodetection of BBS10 in the Bbs10 fl/fl ; Cadh16-Cre +/− tubular region. (A) Representative epifluorescence picture of BBS10-immunostained cryosection of a distal tubule in the medullary region of mice with the indicated genotype. (B) 3D images of immunostained distal tubules against BBS10 for the indicated genotype from 3-month-old male mice generated from a Z-stack of epifluorescence pictures including the image showed in (A). [file 13630_2015_19_MOESM6_ESM.pdf]

Immunodetection of BBS10 in the *Bbs10$^{fl/fl}$; Cadh16-Cre$^{+/-}$* tubular region

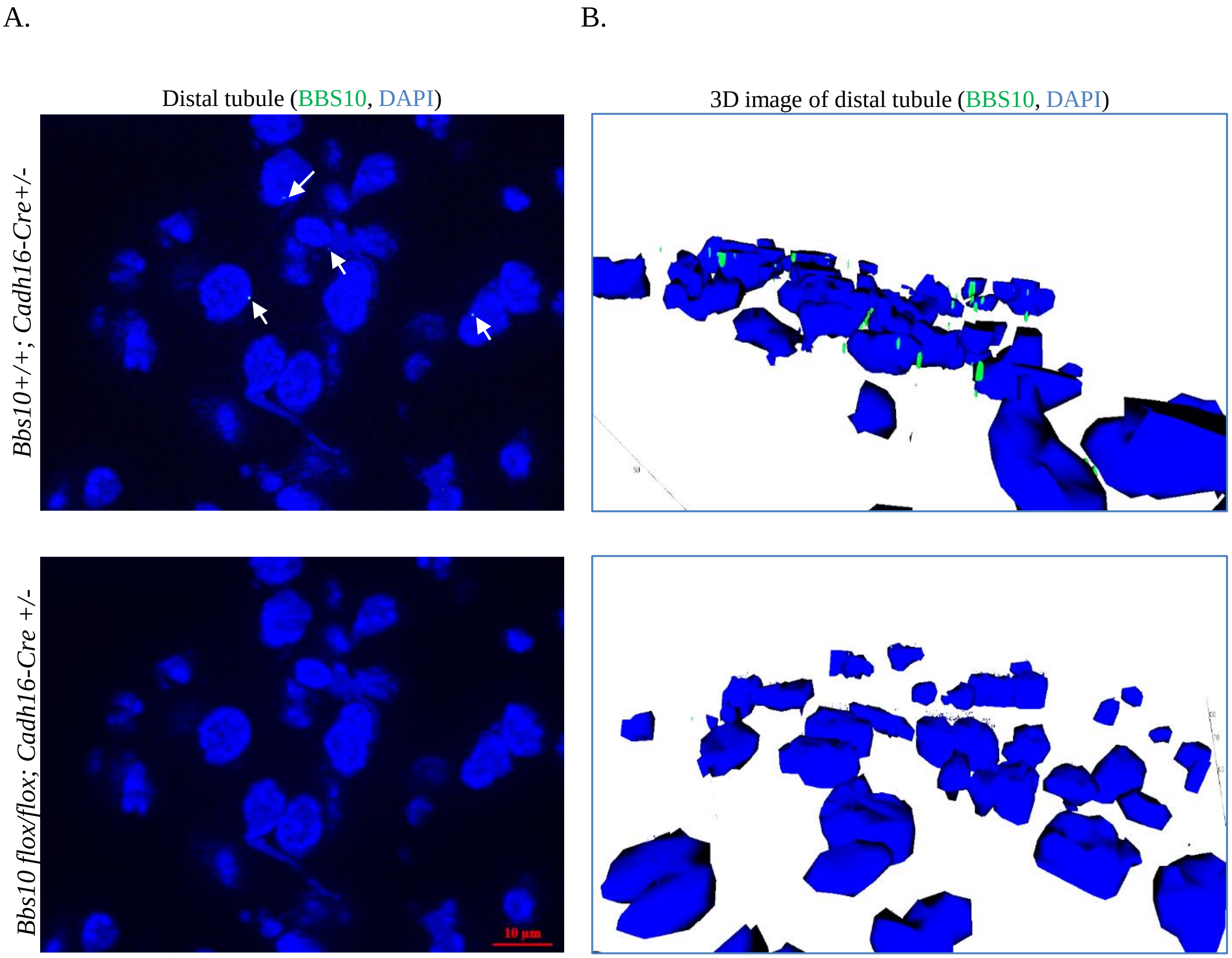

Additional Figure 5
